# Gender-specific Knowledge of Diabetes and Its Management Among Patients Visiting Outpatient Clinics in Faisalabad, Pakistan

**DOI:** 10.7759/cureus.3119

**Published:** 2018-08-08

**Authors:** Ahmad Shahzad, Muhammad Masood Ahmad, Ijaz Anwer, Noor Ijaz, Maheen Shahzad, Muhammad Usman

**Affiliations:** 1 Family Medicine/Diabetology, Al Raheem Clinics, Faisalabad, PAK; 2 Family Medicine/Diabetology, Masood Medicare, Faisalabad, PAK; 3 Family Medicine, Diabetology, Anwer Clinic, Faisalabad, PAK; 4 Demonstrator, College of Medicine and Dentistry University of Lahore, Lahore, PAK; 5 Medical Student, Faisalabad University Medical and Dental College, Faisalabad, PAK; 6 Final Year Medical Student, King Edward Medical University, Lahore, PAK

**Keywords:** diabetes mellitus, t2dm, knowledge, gender, pakistan

## Abstract

Introduction: Diabetes mellitus is an emerging public health concern. The aim of this study was to assess the gender-specific knowledge of patients about diabetes mellitus, its complications, and its management.

Methods: A cross-sectional study was conducted in outpatient clinics of Faisalabad, Pakistan, from November 2017 to March 2018. Consecutive patients with diabetes, aged >18 years, were administered a validated questionnaire related to knowledge of diabetes, its complications, and its management. An analysis was conducted using IBM SPSS Statistics for Windows, Version 19.0 software (IBM Corp., Armonk, NY). Results were stratified on the basis of gender and were compared using chi-square tests.

Results: Of the 840 patients recruited, 76.4% were aged >50 years. About 57% were women, and 43% were men. Most men (89.4%) and women (91.7%) were aware that the management of diabetes requires a cutting down in the consumption of refined sugar, and 64.6% and 50.4%, respectively, reported that they exercise regularly to control their glucose levels. Moreover, 14% of the men and 25% of the women responded that they knew neuropathy is a complication of diabetes.

Conclusion: Diabetes mellitus has debilitating effects on patients and communities. To effectively manage diabetes and to delay the development of complications, there is a dire need to educate patients, families, and communities.

## Introduction

Diabetes mellitus (DM) is an emerging public health concern with multiple complications and an alarmingly increasing prevalence worldwide, especially in the Middle East and South Asia [1]. The International Diabetic Federation (IDF) reported that 415 million people worldwide had DM in 2015 and estimated that 642 million people will have this disease by 2040 [2]. One-third of people with diabetes live in low- and middle-income countries, and approximately 90% have type 2 DM [3-4].

The Diabetes Prevalence Survey of Pakistan 2016-17 revealed that 16.98% of the total population had DM [5-6]. In contrast, the IDF 2017 Atlas estimated that 7.6 million people in Pakistan, or 6.5% of the population, had DM [7].

To decrease the prevalence of this disease, prevention strategies are imperative. Awareness regarding risk factors, such as lifestyle modification and exercise, is crucial [8-9]. Studies have reported that knowledge of and awareness about a disease can have positive effects on patients’ health outcomes, such as increased compliance to the medication and less chances of hospital admission etc. [10-12].

The objective of this study was to assess the gender-specific knowledge about DM, its complications, and management in Pakistan. The findings of this study could help in designing effective gender-specific educational programs for the prevention of this disease.

## Materials and methods

A descriptive cross-sectional study was conducted in the outpatient clinics (OPDs) in Faisalabad, Pakistan from November 2017 to March 2018. Included in this study were diabetic patients aged >18 years who visited the OPDs of the selected family medicine clinics and those who consented to participate in the study. Patients with serious co-morbidities, including cancer or cardiac, respiratory, renal, or liver failure, were excluded. A total of 840 patients were recruited using a non-probability consecutive sampling technique.

The validated questionnaire consisted of three sections: demographic characteristics, knowledge of diabetes, and patients’ self-care management of diabetes; most questions had binary responses (yes/no). Data collectors were trained for obtaining consent and getting the forms filled up.

Written informed consent was obtained from all eligible participants, with patient confidentiality maintained during data collection. The study was conducted in accordance with the code and ethics of the Declaration of Helsinki.

Data were entered and analyzed using IBM SPSS Statistics for Windows, Version 19.0 software (IBM Corp., Armonk, NY). Categorical data were reported as frequencies and proportions. Results were stratified on the basis of gender and were compared using chi-square tests. A p-value <0.05 was considered statistically significant.

## Results

Of the 900 eligible participants who were invited to participate in the study, 840 (93%) provided consent and were included in the final analysis. About 57% were women and 43% were men. Of the study subjects, 76.4% of the patients were aged >50 years, 21% had no formal education, and 21% had completed matriculation. Most of the patients (90.1%) were married, and 50% reported having other co-morbidities (Table 1).

**Table 1:** Socio-demographic characteristics of study participants (N = 840)

Variables	n	%
Age		
< 50 years	198	23.6%
> 50 years	642	76.4%
Educational Status		
Can read or write	176	21.0%
Primary (grades 0–5)	190	22.6%
Secondary (grades 6-9)	106	12.6%
Matriculation (grade 10)	176	21.0%
Intermediate and above	192	22.8%
Marital Status		
Never Married/Widowed	83	9.9%
Married	757	90.1%
Occupational Status		
Employed	392	46.7%
Unemployed/Student/Homemaker	448	53.3%
Comorbidities		
Yes	420	50.0%
No	420	50.0%

Table 2 presents the gender-specific knowledge of patients regarding DM. Approximately 46.5% of the men and 43.5% of the women thought diabetes is a communicable disease. Most of the men (89.4%) and women (91.7%) were aware that the management of DM requires cutting down the consumption of refined sugar, and 76% and 84.6%, respectively, were aware that retinopathy/blindness was a complication of DM.

**Table 2:** Gender-specific knowledge of diabetes mellitus among study participants (N = 840) P value, Pearson chi square p value significant at < 0.05; % (percentage), indicates positive responses to the questions.

Statements	Men (n=359)		Women (n=481)		P-value
	n	%	n	%	
Diabetes mellitus is a curable disease	171	47.6%	251	52.2%	0.192
Diabetes mellitus runs in families	244	68.0%	326	67.8%	0.953
Diabetes is a communicable disease	167	46.5%	209	43.5%	0.377
Management of diabetes mellitus requires cutting down on sweets and refined sugar	321	89.4%	441	91.7%	0.262
Management of diabetes mellitus requires regular physical exercise	303	84.4%	403	83.8%	0.809
Management of diabetes mellitus requires a reduction in body weight in overweight and obese patients	234	65.2%	289	60.1%	0.132
Smoking and tobacco use are more harmful in a diabetic patient	228	63.5%	209	43.5%	0.000
Patients become dependent on oral tablets used for control of blood sugar	83	23.1%	60	12.5%	0.000
Patients become dependent on insulin used for control of blood sugar	99	27.6%	126	26.2%	0.655

Table 3 presents the self-care management practices of diabetes. About 64.6% of men and 50.4% of women reported they exercise regularly to control their blood glucose levels. Moreover, most reported they restrict their intake of sugars and oily foods. Approximately 3.9% of men and 5.4% of women take nonallopathic treatment to help control their DM.

**Table 3:** Self-care management of diabetes among study participants (N = 840) Abbreviation: P value, Pearson chi square p value significant at < 0.05; % (percentage), indicates positive responses to the questions.

Statements	Men (n=359)		Women (n=481)		P-value
	n	%	n	%	
Do you exercise to control your blood sugar?	232	64.6%	243	50.5%	0.000
Do you restrict intake of sweets, sugars and oily foods?	290	80.8%	390	81.1%	0.912
Do you try to lose weight?	186	51.8%	174	36.2%	0.000
Do you take tablets to control diabetes?	271	75.5%	384	79.8%	0.133
Do you take insulin to control diabetes?	175	48.7%	213	44.3%	0.199
Do you visit your doctor regularly for control of diabetes?	253	70.5%	343	71.3%	0.792
Do you self-monitor your blood sugar?	225	62.7%	289	60.1%	0.446
Do you monitor and control serum cholesterol as part of diabetes management?	158	44.0%	176	36.6%	0.030
Do you monitor and control blood pressure as part of diabetes management?	211	58.8%	283	58.8%	0.986
Do you smoke?	135	37.6%	16	3.3%	0.000
If you smoke then have you tried to stop?	62	17.3%	15	3.1%	0.000
Did you give up smoking as part of diabetes management?	51	14.2%	7	1.5%	0.000
Have you taken non-allopathic treatment for control of diabetes?	14	3.9%	26	5.4%	0.312

**Figure 1:**
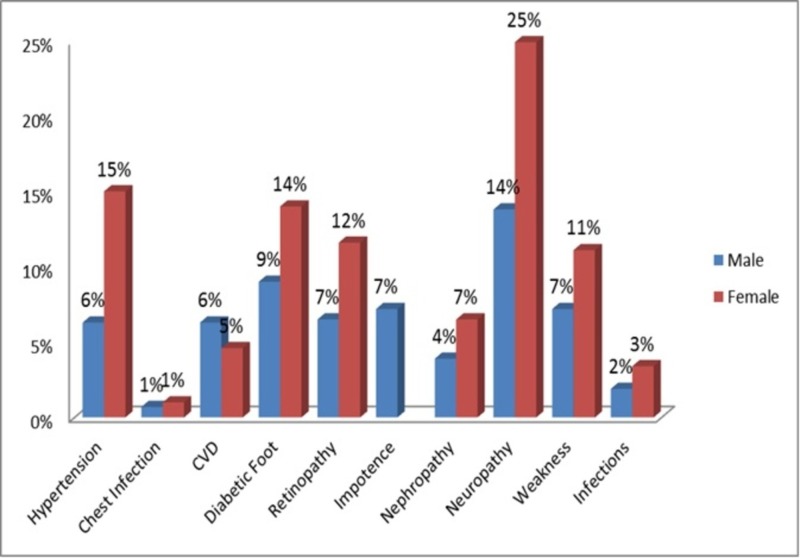
Complications of diabetes among study participants

Figure 1 presents the complications of diabetes among study participants. Overall, 49.3% (n=414) of the patients were suffering from complications of diabetes. Out of these 414 patients, 267 were females and 147 were males. The most common complication was neuropathy (numbness, tingling sensation), which was prevalent in about 25% female and 14% male patients. Moreover, hypertension was prevalent in 15% of the female and 6% of the male study population. About 11% of the female patients responded that they feel generalized weakness and 14% were suffering from diabetic foot.

**Figure 2:**
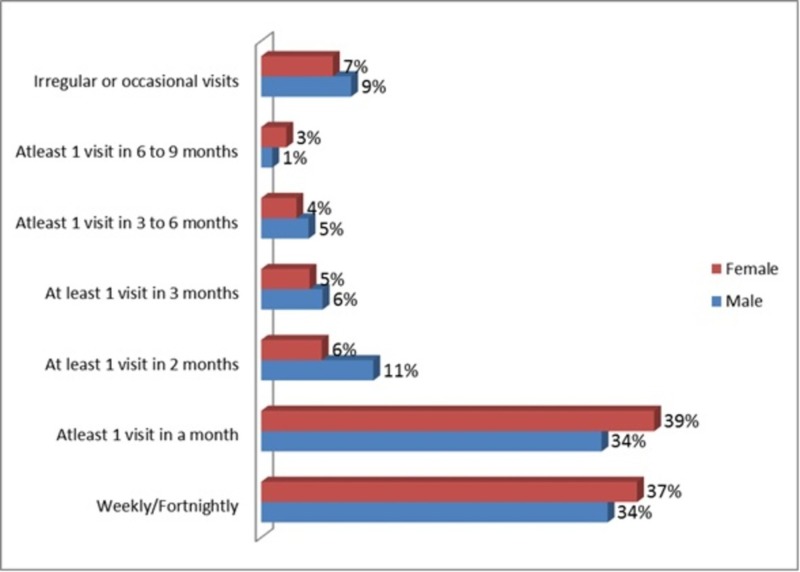
Frequency of patient visit to doctor for diabetes

Figure 2 depicts the frequency of patients’ visiting doctors clinics regarding diabetes. About 37% female patients and 34% male patients visited doctors weekly or fortnightly, while 6% male and 5% female patients visited the doctor at least once in three months.

## Discussion

The proper management of DM is challenging, especially for people living in developing countries, due to the lack of access and the affordability of health care services. The IDF and American Diabetes Association have stressed the importance of clinical care, self-care practices, and patient education in the management and prevention of complications associated with DM [13-16]. These patients should have a working knowledge of diabetes, including its signs and symptoms, as well as diabetes care and management [17-18]. This study assessed the gender-specific knowledge of diabetic patients about DM and its complications and management.

The current study found no major differences between men and women regarding their basic knowledge of DM and its management. In contrast, previous studies found that men were significantly less knowledgeable about diabetes than women [19-21]. In the current study, women were found to be less knowledgeable than men regarding the harmful effects of smoking and tobacco in diabetic patients, perhaps because smoking by Pakistani women is uncommon. Nevertheless, diabetic patients should be informed about the harmful effects of chewing tobacco, in the form of paan or betel nuts, which is much more prevalent among Pakistani women [22-23].

The current study reveals that most patients were aware that an unhealthy diet and refined sugar are risk factors for complications of DM. These results are similar to those reported by a population-based study in Pelotas, South Brazil [24].

Physical activity can also reduce the likelihood of the complications of DM. A quasi-experimental study in 65 patients with type 2 DM reported that an exercise regime longer than 12 weeks was associated with reductions in glycosylated hemoglobin levels in diabetic patients [25]. Our study found that 84% of men and 83% of women patients were aware that regular exercise is necessary to maintain optimum glycemic levels.

The complications of diabetes are associated with morbidity, mortality, and increased health care costs [26]. Patients in this study were aware, in descending order, of the following complications of DM: retinopathy/blindness, amputations, heart diseases, neuropathy, nephropathy, and infections. A study conducted in Peshawar, Pakistan, also reported similar results [27].

The study had certain limitations. This was a descriptive cross-sectional study so there is a need for longitudinal studies to assess temporal associations between gender and knowledge of diabetes. Moreover, a reporting bias could have occurred in this study as we relied on the self-reporting of the patients about the co-morbidities and complications of diabetes. This may have over-estimated or under-estimated the study results. We also did not ask participants regarding the optimum level of glycemic control, which could have given us more information about their knowledge regarding DM. Additionally, it will be interesting to see a comparison between urban and rural areas of Pakistan so that a community-based diabetes awareness program could be implemented on a national scale.

## Conclusions

Diabetes mellitus has one of the most debilitating effects on the patient and the community owing to the disease itself and its complications. We found that though there was not a notable difference in the knowledge levels of the male and female population except for one or two aspects of diabetes, there are many patients who lack awareness and basic knowledge of the complications of this disease.

In order to manage DM effectively and to delay the development of complications, there is a need to educate the patient, family, and community. The health care providers should organize health education sessions to provide early diabetic education regarding causes, self- management, lifestyle modifications, and preventive measures for diabetic complications. This could help in diabetes management and will, in turn, improve the quality of life of the patients. Moreover, the formation of a diabetes support group can also be very beneficial for the patient and their families. This study also found that some patients were following good practices. Such patients should be kept motivated so that they may continue their health routine and can become a source of inspiration to other patients.
